# Sense of purpose and perception of involution (*Nei Juan*): the role of actual-ideal self-discrepancy and future time perspective

**DOI:** 10.3389/fpsyg.2026.1891243

**Published:** 2026-08-07

**Authors:** Guoliang Yang, Zhihua Wang, Miaoying Fang, Weijiong Wu, Ruiming Wang

**Affiliations:** 1 School of Digital Economics and Trade, Guangzhou Maritime University, Guangzhou, China; 2 School of Management, Wuzhou University, Wuzhou, China; 3 School of Management, Guangdong University of Technology, Guangzhou, China; 4 Center for Studies of Psychological Application, South China Normal University, Guangzhou, China

**Keywords:** actual-ideal self-discrepancy, de-involution, future time perspective, involution perception, sense of purpose

## Abstract

**Objective:**

The concurrent increase in “hollowed-heart” syndrome (which describes a state of purposelessness) and involution anxiety severely impairs the psychological development of contemporary Chinese undergraduates. From a de-involution perspective, this study aimed to examine the relationship between sense of purpose and perceived involution, and to explore the mediating role of actual-ideal self-discrepancy and the moderating effect of future time perspective in this association.

**Method:**

A total of 747 Chinese undergraduates completed online and offline questionnaires measuring the four core psychological variables.

**Results:**

(1) Sense of purpose exerted a significant negative direct effect (β = −0.27, *p* < 0.001) on perceived involution; (2) Actual-ideal self-discrepancy partially mediated this negative relationship (indirect effect = −0.06, SE = 0.01, 95% CI [−0.08, −0.03]); (3) Future time perspective moderated the first-stage path of the indirect effect. Simple slope analyses showed that the sense of purpose–perceived involution association was significant at a low (*M* − 1*SD*) level of future time perspective (simple slope: β = −0.24, *p* < 0.001), but was not significant at a high (*M* + 1*SD*) level of future time perspective (simple slope: β = −0.02, *p* > 0.05), such that the mediating role of actual-ideal self-discrepancy was weaker among individuals with a higher future time perspective.

**Conclusion:**

A stronger sense of purpose mitigates undergraduates’ perceived involution by reducing actual–ideal self-discrepancy. Cultivating life purpose and future-oriented cognition may help alleviate undergraduates’ involution anxiety. Based on self-discrepancy theory and socioemotional selectivity theory, these findings clarify the underlying mechanism of the involution perception and have practical implications for college mental health education and targeted stress interventions.

## Introduction

The imperative to mitigate involution–which has been referred to as “de-involution”–has emerged as a pressing concern across numerous Asian countries, including China, as well as certain Western societies (Xu et al., 2025). Involution, which is called “*Nei Juan*” (“内卷”) in Chinese, is characterized by societal hypercompetition and intensified individual effort that does not result in proportional gain (Zhang et al., 2025) and engenders not only negative psychological states such as anxiety, burnout, and hopelessness but also maladaptive behavioral responses like “goblin mode” (“摆烂,” Cao and Chen, 2023; Chang and Wu, 2026). However, there have been few studies that have examined how individuals can attain a state of genuine de-involution independently of these macro-level factors. To lay a theoretical foundation for addressing this research gap, we first clarify the core conceptual connotations of involution and de-involution.

Involution originally referred to “growth without development” through excessive refinement (Xu et al., 2025; Zhang et al., 2024). It now denotes the phenomenon in which individuals, when confined to resource-limited environments, exhibit adverse psychological states and non-benign competitive behaviors (Huang, 2021; Zhang et al., 2024, 2025). Similarly, de-involution serves as the counterpart to involution and generally refers to resolving or controlling the causes of involution (Huang, 2021; Tong et al., 2024; Xu et al., 2025). The related discourse has been focused on improving external conditions (e.g., transforming growth models and fostering diverse cultures) and supporting internal mentalities (e.g., modifying self-perception and rationally planning developmental trajectories) (Zhang and Feng, 2021; Zhang and Wu, 2022; Wang et al., 2025). However, the literature tends to emphasize macro-level regulation and theoretical exposition, which poses challenges to implementation at the individual micro level. In the present study, the coexistence of hollow-heart phenomena (Ni and Shen, 2025; Xie and Zhang, 2022) and involution anxiety among contemporary college students is addressed; the ways that individuals’ sense of purpose influences their involution experiences are investigated. Critically, this study is aimed at proposing microlevel psychological adaptive strategies for de-involution.

A sense of purpose refers to an individual’s feelings of meaning, direction, and purpose in life (Ryff, 1989; Hill et al., 2022). As a means of addressing the macrolevel attributes of the causes of involution and its consequent negative impacts, a sense of purpose can, based on the social-emotional-behavioral skills framework (Soto et al., 2022, 2024; Beatty et al., 2025), assist college students in value introspection and adaptive adjustments, thereby promoting healthy growth and facilitating the construction of a de-involution state. A large body of psychological and sociological literature identifies involution-related anxiety as the core component or representative manifestation of perceived involution (Fu, 2022; Xu et al., 2025). Consistently, existing countermeasures against involution suggest that individuals should maintain a rational mindset, objectively evaluate online discourses about involution, and form unbiased perceptions of their own capabilities and ideal status to alleviate involution-induced anxiety (Cao and Chen, 2023; Tong et al., 2024). Taken together, involution-related anxiety lies at the heart of perceived involution in both its formation mechanisms and targeted interventions. Therefore, this study adopts involution anxiety as the primary proxy for perceived involution.

Involution reflects the deleterious impact of resource scarcity and homogenized competition on individuals and manifests at the micro level as involution anxiety stemming from the discrepancy between one’s ideal and actual selves (Tong et al., 2024). Sociological research supports the positive role of actual-ideal self-discrepancy in shaping involution perception: the divergence between societal expectations and reality affects young people’s cognitive systems, thereby engendering psychological dissonance and self-denial regarding existential meaning and value and fostering modernity anxiety in society (Fu, 2022; Lian, 2025). According to self-discrepancy theory (Higgins, 1987; Kee and Neshteruk, 2026), the ideal self refers to the attributes or characteristics that an individual aspires to possess; the actual self denotes the attributes or characteristics that an individual actually possesses. Within the current environment, “once a disconnect emerges between expectations and reality, it generates intense psychological dissonance and a sense of relative deprivation” (Lian, 2025). Hence, actual-ideal self-discrepancy intensifies an individual’s perception of involution (Tong et al., 2024). Conversely, according to the sense of purpose theory, a sense of purpose guides individuals to engage in both daily and long-term goals, provides meaning and rigor to life, and thereby serves as a regulator for maintaining self-balance and directing daily life (Burrow et al., 2024). These propositions are aligned with positive coping strategies in self-discrepancy theory, which are aimed at narrowing the gap between our actual and ideal selves (Liw and Han, 2022; Wang et al., 2024).

Both social-cognitive and goal-based motivational theories conceptualize the future time perspective (FTP) as a foundational determinant of behavior (Kooij et al., 2018; Cai et al., 2026). FTP is defined as individuals’ cognitive, affective, and behavioral orientation toward the future (He et al., 2026). An FTP is a relatively stable personality trait that reflects cognitive, emotional, and behavioral propensities in the anticipation, planning, and construction of possibilities for the future development of both the self and society (Song, 2004; Lyu and Huang, 2016; He et al., 2026). Individuals with high-level FTPs tend to prioritize long-term goals and distal outcomes over immediate or proximal results (Zimbardo and Boyd, 1999; Yang Q. et al., 2021; He et al., 2026). According to the socioemotional selectivity theory, those with high-level FTPs are better equipped to set future goals that can regulate their present behaviors (Carstensen, 2006; Hill et al., 2025); thereby, a high-level FTP amplifies the negative relationship between a sense of purpose and negative affect (Pfund et al., 2022). Conversely, Yang X. J. et al. (2021) identified an antagonistic interaction in which an FTP attenuated the negative relationship between the protective factor (mindfulness) and the negative outcome (boredom). Given these divergent views on the moderating role of FTP, the present study refrains from presenting *a priori* specifications of the direction or magnitude of this moderation. The specific direction of this moderating effect is further examined through theoretical analysis grounded in contextual considerations.

The present study is aimed at addressing the aforementioned gap by investigating three central questions: (1) Can enhancing their sense of purpose contribute to individuals’ perception of involution in their current environment? (2) What role does the actual-ideal self-discrepancy play in this process? (3) What de-involution process changes occur as a result of changing one’s sense of purpose when the FTP is different? Based on the theoretical framework and the above research questions, the following hypotheses are proposed in this study. Hypothesis 1: A sense of purpose negatively links to an individual’s perception of involution. Hypothesis 2: A sense of purpose is negatively associated with the perceptions of involution through the mediating effect of actual-ideal self-discrepancy. Hypothesis 3: The indirect effect of a sense of purpose on involution perception, as mediated by actual-ideal self-discrepancy, is moderated by FTP.

## Materials and method

### The present study

**FIGURE 1:**
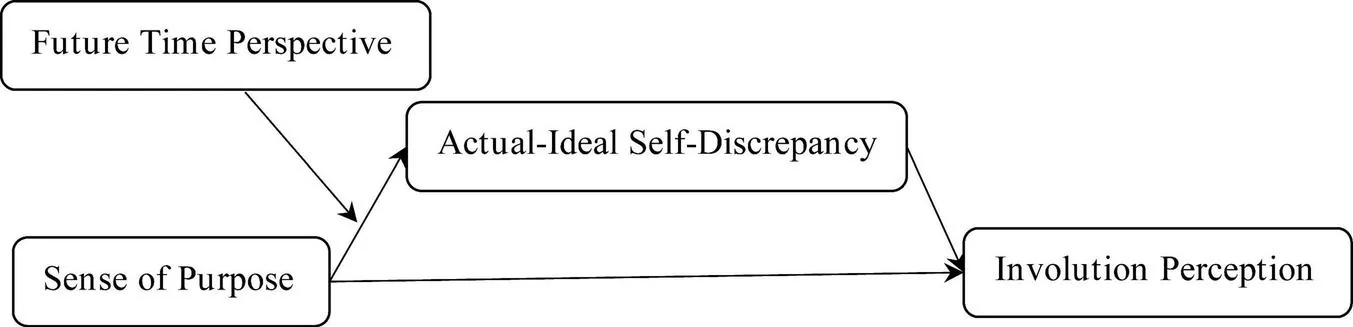
Conceptual model. Source: Authors’ own work.

Grounded in a de-involution perspective and integrating the social-emotional-behavioral skills framework with self-discrepancy theory, a moderated mediation model (see Figure 1) is proposed for investigating the relationships among a sense of purpose, FTP, actual-ideal self-discrepancy, and involution perception. Specifically, the mediating mechanism (actual-ideal self-discrepancy) and the moderating effect (FTP) through which sense of purpose predicts individuals’ perceptions of involution are examined in this study while controlling for sex and age. This research is aimed at providing empirical evidence and theoretical guidance for de-involution efforts among college students navigating contemporary *Nei Juan* environments.

### Participants

The survey targeted undergraduate students and employed a mixed-mode data collection strategy that combines online and offline methods. After excluding invalid responses, a total of 747 valid questionnaires were retained. Cluster sampling among the undergraduates at two universities in Guangdong and Guangxi Provinces, China, was utilized for offline sampling, yielding 292 valid questionnaires (for a 97.33% response rate). A total of 190 valid questionnaires were subsequently collected through random campus intercept interviews (for a 95% response rate). Online distribution via the Credamo platform–a professional research data platform–generated 265 valid responses (94.64% response rate), with participants’ IP addresses spanning 150 universities nationwide. Heterogeneity tests revealed no significant differences among the three groups in regard to the “intensity of *Nei Juan* competition ratings” (*F* = 1.87, *p* > 0.05), which indicates homogeneity across the sampling methods. The sample included 412 males (55.2%) and 335 females (44.8%). The class standing distribution was as follows: freshmen, 190 (25.43%); sophomores, 90 (12.05%); juniors, 237 (31.73%); seniors, 214 (28.65%); and fifth-year students (e.g., medical majors), 16 (2.14%).

### Measures

#### Sense of purpose

The revised 14-item Sense of Purpose Scale (Sharma and Yukhymenko, 2019; Cai et al., 2026) was adopted for this study. A representative item is “My purpose in life is clear.” The participants rated each statement on a 5-point Likert scale (1 = strongly disagree to 5 = strongly agree), where higher mean scores indicate a stronger sense of purpose. The Cronbach’s α for this scale was 0.927.

#### Actual-ideal self-discrepancy

The Integrated Self-Discrepancy Index (Hardin and Lakin, 2009; Gürcan-Yıldırım and Gençöz, 2022) was utilized for this study. The participants first generated five words that best described their personality traits. Following an explanation of the ideal self-construct, participants rated the extent to which they believed that their ideal self possessed each of the five listed traits (1 = not at all present to 5 = completely present). The concept of the actual self was subsequently explained, and the participants rated the degree to which their actual self was in possession of each of these five traits. The total actual-ideal self-discrepancy score was calculated by subtracting the actual self-score from the ideal self-score and taking the absolute value of the difference, with higher scores indicating a greater discrepancy. The Cronbach’s α reliabilities for the ideal self-scale and actual self-scale were 0.773 and 0.837, respectively, which supports the calculation of the actual-ideal self-discrepancy index.

#### Future time perspective

The Future Time Perspective Scale for Chinese college students (Song, 2004; Liu et al., 2026) was adopted for this study. This 20-item scale includes statements such as “I have many things I want to do in the future” and “I often imagine how I will change in the course of my future life.” Items were scored on a 4-point scale (1 = strongly agree to 4 = strongly disagree), with the reverse-scored items recoded such that higher scores indicate a stronger FTP. For the present sample, the internal consistency of this scale was Cronbach’s α = 0.900.

#### Involution perception

The five-item anxiety subscale of the GHQ-20 (Li and Mei, 2002; Li and Lin, 2003; Li W. et al., 2024) was employed in this study. The items were scored dichotomously (0 = absent and 1 = present), with higher total scores indicating more severe perceptions of involution. The Cronbach’s α for this scale was 0.909. The decision not to adopt the involution perception scale developed by Zhang et al. (2024) was based on three considerations: First, two components in the original scale explicitly conceptualize “resource scarcity” and “social norms” as situational and organizational conditions of involution, respectively, and position them as antecedent factors rather than as constituents of the construct *per se*; second, some dimensions such as “individual psychology” and “group behavior, culture, and social ecology” of the four-dimensional framework may not be located at their commensurate conceptual levels; and third, those items used to measure “psychological pressure” in the original scale (e.g., “feeling down,” “nervous tension”) demonstrate substantial conceptual overlap with the anxiety items (e.g., “restlessness and tension,” “mental stress”) used in the GHQ-20. Furthermore, two contextual priming items were administered before participants completed the anxiety subscale to anchor their affective responses to the specific involution context. Respondents first rated their familiarity with prevalent societal involution phenomena on a 5-point Likert scale (1 = extremely unfamiliar to 5 = extremely familiar; *M* = 3.85, SD = 0.03), which serves as a contextual priming manipulation (Cui et al., 2026). Immediately afterward, participants reported the perceived severity of social involution on a 0–100 sliding scale, with higher scores indicating stronger involution perceptions (*M* = 75.67, SD = 0.56). Thanks to this sequential priming focusing on involution familiarity and severity, the anxiety captured by the GHQ-20 subscale specifically reflects context-bound involution perceptions rather than generic, non-specific anxiety symptoms.

#### Data collection and analysis

This study received ethics approval from the university ethics committee, and informed consent was obtained from each of the participants. Data collection occurred in three phases: In Phase I, cluster sampling was employed across two universities; in Phase II, online data collection was conducted via the Credamo platform; and in Phase III, random campus intercept interviews were employed. Data analysis was conducted using SPSS 26.0 and PROCESS macro version 3.5 (Hayes, 2013; Hong et al., 2026).

## Results

### Common method bias

Two approaches were implemented to minimize common method bias. During data collection, the procedural remedies included reverse-coded items, anonymous responses, and randomized item order (Podsakoff et al., 2024). During the analysis phase, Harman’s single-factor test and the CFA-based unmeasured latent variable (UMLV) technique (Podsakoff et al., 2024; Zhou and Long, 2004) were conducted. The Harman’s test indicated that eight factors with eigenvalues of greater than 1 were extracted without rotation, with the first factor accounting for 29.23% of the total variance–which is below the critical threshold of 40%–suggesting that common method bias was not a significant concern in this study. Furthermore, the CFA-based UMLV approach demonstrated that the model fit indices did not substantially improve upon the inclusion of a latent method factor [ΔCFI = 0.09, ΔRMSEA = 0.015, ΔTLI = 0.084; from the CFA Model (χ^2^/*df* = 7.319, CFI = 0.743, TFI = 0.726, RMSEA = 0.092) and CFA-ULMV model (χ^2^/*df* = 5.366, CFI = 0.833, TFI = 0.810, RMSEA = 0.077)]. According to established conventions, changes in fit indices of ΔCFI < 0.1 and ΔRMSEA < 0.05 indicate that common method bias is not a serious concern (Podsakoff et al., 2024). Collectively, these results suggest that CMB is unlikely to be a serious threat to the validity of our findings.

### Descriptive statistics and correlation analysis

The descriptive statistics and correlation coefficients are presented in Table 1. Both age and sex were positively correlated with sense of purpose and were therefore included as covariates in the subsequent analyses. As hypothesized, both a sense of purpose and FTP were negatively correlated with actual-ideal self-discrepancy and involution perception, whereas actual-ideal self-discrepancy was positively correlated with involution perception.

**TABLE 1:** Descriptive statistics and bivariate interrelations (*n* = 747).

Variables	*M*	*SD*	1	2	3	4	5
1. Sex	0.55	0.50	0.11**	0.30***	0.65***	−0.16***	0.31***
2. Age	20.77	1.58					
3. SOP	3.60	0.71	0.08*				
4. FTP	2.93	0.46	−0.07	0.26***			
5. AISD	3.84	3.44	0.04	0.05	−0.18***		
6. IP	1.47	1.70	−0.04	0.08*	−0.22***	−0.21***	

SOP, sense of purpose; FTP, future time perspective; AISD, actual-ideal self-discrepancy; IP, involution perception; Sex was dummy-coded as female = 0 and male = 1; and

*p < 0.05,

**p < 0.01, and

***p < 0.001.

### Moderated mediation model test

The variable data were standardized, and PROCESS Model 4 (with 5000 bootstrap resamples) was used to examine the relationship between a sense of purpose and involution perception, with actual-ideal self-discrepancy specified as the mediating variable. The results (Table 2, Model 1) indicate that after controlling for sex and age, the main effect of a sense of purpose on perceived involution was significant (β = −0.27, *t* = −7.26, *p* < 0.05, 95% CI [−0.34, −0.20]), which indicates that a sense of purpose negatively predicted the perception of involution, thereby supporting Hypothesis 1.

**TABLE 2:** Testing the moderated mediation model.

Variables	IP	AISD	IP	AISD
	Model 1	Model 2	Model 3	Model 4
	β 95% CI	β 95% CI	β 95% CI	β 95% CI
Sex	−0.04 [−0.11,0.03]	−0.05 [−0.12, 0.02]	−0.03 [−0.10,0.04]	−0.05 [−0.12,0.02]
Age	0.15*** [0.08,0.23]	−0.05 [−0.12, 0.02]	0.13*** [0.05, 0.20]	0.08* [0.01, 0.16]
SOP	−0.27*** [−0.34, −0.20]	−0.21*** [−0.29, −0.14]	−0.21*** [−0.29, −0.14]	−0.13* [−0.23, −0.04]
AISD			0.26*** [0.19, 0.33]	
FTP				−0.10* [−0.19, −0.01]
SOP × FTP				0.11*** [0.05, 0.17]
*R* ^2^	0.07	0.04	0.14	0.06
*F*	19.37	11.44	29.79	10.22

SOP, sense of purpose; FTP, future time perspective; AISD, actual-ideal self-discrepancy; IP, involution perception; Gender was dummy-coded as female = 0 and male = 1; and

*p < 0.05 and

***p < 0.001.

The results of the mediation analysis (Table 2, Models 2 and 3) revealed that after controlling for sex and age, when the actual-ideal self-discrepancy was simultaneously entered into the model to predict involution perception, the direct effect of a sense of purpose remained significant but diminished (β = −0.21, *t* = −5.84, *p* < 0.05, 95% CI [−0.29, −0.14]). A sense of purpose significantly predicted actual-ideal self-discrepancy (β = −0.21, *t* = −5.63, *p* < 0.05, 95% CI [−0.29, −0.14]), and actual-ideal self-discrepancy significantly and positively predicted involution perception (β = 0.26, *t* = 7.53, *p* < 0.05, 95% CI[0.19, 0.33]). These findings indicate that actual-ideal self-discrepancy partially mediated the relationship between sense of purpose and involution perception (indirect effect = −0.06, SE = 0.01, 95% CI [−0.08, −0.03]), thereby supporting Hypothesis 2.

The moderating effect of an FTP was examined using PROCESS Model 7 (5000 bootstrap resamples). The results (Table 2, Model 4) indicate that the interaction between a sense of purpose and FTP significantly predicted actual-ideal self-discrepancy (β = 0.11, *t* = 3.44, *p* < 0.05; 95% CI [0.05, 0.17]), which demonstrates that the indirect effect of a sense of purpose on involution perception via actual-ideal self-discrepancy was moderated by FTP. Thus, Hypothesis 3 is supported.

**FIGURE 2:**
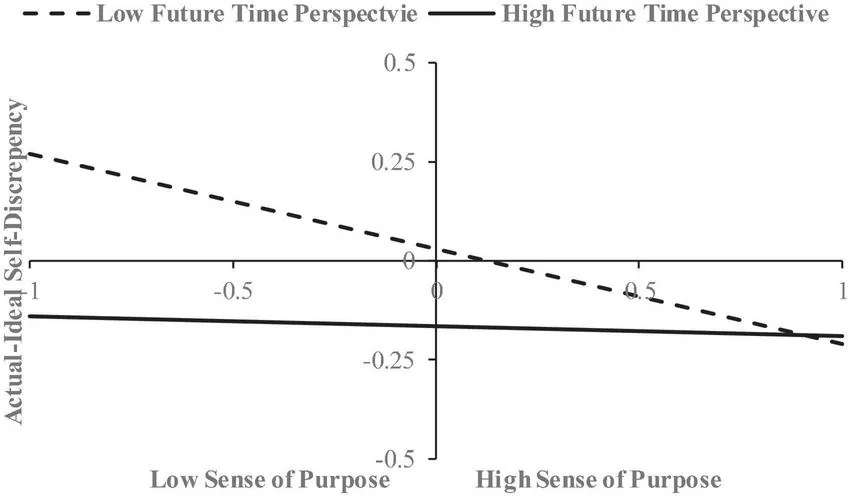
The moderating role of future time perspective in the relationship between a sense of purpose and actual-ideal self-discrepancy. Source: Authors’ own work.

Furthermore, we conducted a simple slope test by categorizing participants into high- and low-level FTP groups based on the mean (*M*) ± 1 standard deviation (*SD*). The results (Figure 2) reveal that for participants with lower-level FTPs, a sense of purpose significantly and negatively predicted actual-ideal self-discrepancy (βsimple = −0.24, *t* = −4.47, *p* < 0.05). For participants with higher-level FTPs, the negative predictive effect of a sense of purpose on actual-ideal self-discrepancy was no longer significant (βsimple = −0.02, *t* = −0.41, *p* > 0.05). This pattern indicates that as the level of FTP increases, the negative relationship between a sense of purpose and actual-ideal self-discrepancy attenuates. Furthermore, at the low (*M*−1*SD*), moderate (*M*), and high (*M* + 1*SD*) levels of FTP, the conditional indirect effect values and 95% confidence intervals for actual-ideal self-discrepancy were −0.06 [−0.10, −0.03], −0.03 [−0.07, −0.01], and −0.01[−0.04, 0.02], respectively, which indicates that the mediating effect of actual-ideal self-discrepancy in the relationship between a sense of purpose and involution perception gradually decreases as the level of FTP increases.

## Discussion

Grounded in the social-emotional-behavioral skills framework and self-discrepancy theory and incorporating socioemotional selectivity theory, a moderated mediation model was constructed for this study to elucidate the mechanism through which a sense of purpose influences undergraduates’ perceptions of involution via actual-ideal self-discrepancy, with FTP used as a moderator. The findings contribute to a deeper understanding of how a sense of purpose attenuates involution perception, the mediating function of actual-ideal self-discrepancy in this relationship, and the moderating role of FTP on the association between a sense of purpose and actual-ideal self-discrepancy. These insights hold theoretical and practical significance for effectively addressing involution anxiety and preserving mental health in contexts where the hollowed-heart and *Nei Juan* phenomena coexist.

First, this study supports the proposition that a sense of purpose serves as a protective factor against involution such that a higher sense of purpose is associated with a lower perception of involution (supporting Hypothesis 1), which is consistent with recent scholarship (Hill et al., 2022; Pfund et al., 2024). These findings also provide empirical validation for academic involution research that addresses mitigation strategies targeting “students’ individual actions” (Wang et al., 2025). The protective role of a sense of purpose against involution perception can be explained through the social–emotional–behavioral skills framework (Soto et al., 2022; Beatty et al., 2025), in which a sense of purpose is posited to help college students develop three types of skills for achieving adaptive outcomes. For instance, a sense of purpose promotes more positive social relationships and enhances both social engagement and collaborative skills. It also helps individuals maintain emotional stability, experience weaker stress responses, and show greater emotional resilience, particularly under pressure.

Furthermore, a sense of purpose enables more effective resource-allocation decisions and the capacity for organizing one’s life around meaningful goals, thereby fostering self-management skills. These three types of skills represent abilities that individuals can autonomously activate within their given environmental conditions to enhance peer acceptance, academic engagement, and adaptive functioning (Soto et al., 2024). In addition, individuals with a strong sense of purpose consciously redirect attention toward their most salient and imminent goals, engage with life more actively, and better commit to future planning, thereby maintaining personal equilibrium (Hill et al., 2016; Burrow et al., 2024). Consequently, when confronted with identical *Nei Juan* conditions, individuals with a robust sense of purpose perceive lower levels of involution pressure.

### Mediating role of actual-ideal self-discrepancy

This study reveals that a sense of purpose not only directly influences individuals’ perceptions of involution but also indirectly affects their actual-ideal self-discrepancy. That is, this discrepancy partially mediates the relationship between a sense of purpose and involution perception (thereby supporting Hypothesis 2). This conclusion provides empirical support for the analytical framework of “social expectations and reality gap - >actual-ideal self-discrepancy - >relative deprivation and social anxiety” used in research on Chinese youth social mentality (Lian, 2025). It also offers empirical evidence for the individual-level “self-cognition - >psychological resilience and emotional regulation - >academic involution” mitigation strategy in the generative mechanism model of academic field-specific involution among undergraduates (Wang et al., 2025). Given the paucity of comparable studies that employ actual-ideal self-discrepancy as a mediating variable–which mostly originate from the social media usage domain (Ahadzadeh et al., 2017; Zhao and Li, 2026)– a focal theoretical model is proposed in the present study through the integration of opinions regarding the generation mechanisms of negative social mentality among youth (Lian, 2025), the generative mechanism model of academic involution among college students (Wang et al., 2025), and the competition pressure-psychological compensation strategy model (Wang et al., 2024). The findings support a partial mediating effect of actual-ideal self-discrepancy on the relationship between a sense of purpose and involution perception. This finding indicates that, beyond external conditions such as resource scarcity, the discrepancy between an individual’s ideal and actual selves functions as a bridging variable that links a lack of purpose to involution anxiety, thereby providing new evidence to support the expansion of self-discrepancy theory to enrich de-involution research.

First, a sense of purpose negatively predicted actual-ideal self-discrepancy, thereby corroborating the claims of Beatty et al. (2025) and Pfund et al. (2024) that a sense of purpose exerts a protective effect on the development of self-management skills. Specifically, such functions as cognitive reconstruction, emotion regulation, and self-equilibrium align with task-oriented coping (Liw and Han, 2022) and direct resolution strategies (Wang et al., 2024) embraced by self-discrepancy coping approaches. Thus, a sense of purpose has a positive effect similar to that of proactive coping in that it is negatively associated with actual-ideal self-discrepancy. This study extends the protective function of a sense of purpose to the domain of self-discrepancy management, supplementing and expanding upon the work of Beatty et al. (2025). Second, this study reveals that individuals’ actual-ideal self-discrepancy significantly and positively predicts their involution perception, which is consistent not only with prior studies on the relationship between actual-ideal self-discrepancy and subsequent negative outcomes (Higgins, 1987; Hu et al., 2022) but also with the assertion presented in sociological studies that “the gap between expectations and reality leads to psychological pressure and involution anxiety” (Fu, 2022; Zhang et al., 2024; Lian, 2025). The external causes of involution and its negative effects stem from resource scarcity and homogenized competition, whereas its internal causes reside in individuals’ actual-ideal self-discrepancies and cognitive dissonance. Extensive prior research in the self-discrepancy literature has confirmed the positive association between actual-ideal self-discrepancy and its consequential outcomes, including anxiety, depression, psychological stress, and moral injury (Higgins, 1987; Liw and Han, 2022; James et al., 2024). Therefore, within the current social environment, the greater the gap between an individual’s ideal self and actual self is, the stronger their perception of involution. From the positive valence analysis of a sense of purpose, and under equivalent conditions, individuals with a higher-level sense of purpose have less actual-ideal self-discrepancy and, consequently, experience a lesser perception of involution.

### Moderating effect of future time perspective

In this study, a moderated mediation model based on the temporal attributes of sense of purpose and the relevant theories of FTP was constructed. It was used to examine the moderating effect of FTP on the mediating role of actual-ideal self-discrepancy in the relationship between a sense of purpose and involution perception. The results indicate that an FTP moderated the indirect effect of a sense of purpose on involution perception through actual-ideal self-discrepancy (thereby supporting Hypothesis 3). Specifically, an FTP moderated the first stage of the mediation model, namely, the relationship between a sense of purpose and actual-ideal self-discrepancy. This moderation manifested as a gradual weakening of the negative association between a sense of purpose and actual-ideal self-discrepancy among individuals with high-level FTPs compared with the large increase and decrease that was observed among those with low-level FTP; thus, this moderation smoothed the negative impact of a sense of purpose on involution perception. These findings align with those of Yang X. J. et al. (2021) and confirm that, in this study, the moderating effect of an FTP is consistent with an antagonistic interaction between the two protective factors (Cohen et al., 2003; Zhang et al., 2023). The absence of a synergistic interaction effect for FTP on the relationship between a sense of purpose and actual-ideal self-discrepancy can be analyzed from two perspectives. First, socioemotional selectivity theory (Carstensen, 2006; Hill et al., 2025) posits that FTP determines the goal selections, preferences, and behaviors of individuals. Unlike those with low-level FTPs who prioritize present experiences and socioemotional goals, individuals with high-level FTPs place greater focus on future opportunities and knowledge-acquisition goals. When confronted with the same *Nei Juan* environment, individuals with low-level FTPs are more susceptible to immediate contextual influences through their prioritization of short-term goals and avoidance of negative experiences (Pfund et al., 2022). Consequently, their sense of purpose has a stronger negative predictive effect on actual-ideal self-discrepancy. Conversely, individuals with high-level FTPs prioritize future opportunities and long-term planning and have more positive perspectives on the future (Allemand et al., 2021; Liu et al., 2026). Therefore, their sense of purpose has a weaker negative predictive effect on their actual-ideal self-discrepancy. Second, the dual motive model of self-control (Dreves and Blackhart, 2019; Rogojina et al., 2025) proposes that an FTP influences self-control by adjusting the relative prioritization of proximal and distal motives. The pursuit of multiple goals is universal. When proximal and distal motivations compete, any factor that increases the incentive value of distal goals or decreases that of proximal goals enhances self-control. Because a high-level FTP facilitates the improved prediction of distal action consequences, it increases the incentive value of long-term goals. Consequently, individuals with high-level FTPs prioritize long-term goals over immediate motives, which results in greater self-control. This enables such individuals to more effectively balance the relationship between their sense of purpose and actual-ideal self-discrepancy, thereby smoothing the steep but potentially damaging decline in perceived involution.

Notably, regarding the moderating effect of FTP, the present study is aligned with that of Yang X. J. et al. (2021) but differs from that of Pfund et al. (2022). This inconsistency may stem from cultural differences (Komatsu et al., 2026; Wang and Liu, 2025) on the one hand and sample age characteristics on the other. Both the present study and that of Yang X. J. et al. (2021) employed samples of domestic university students whose mean ages did not exceed 25 years and were embedded in a long-term oriented cultural context that prioritizes forward-looking pragmatism and long-run planning (Komatsu et al., 2026), whereas Pfund et al. (2022) selected three samples of adults from short-term oriented cultural environments, where individuals tend to prioritize instant rewards and immediate gratification (Wang and Liu, 2025); moreover, whose mean ages were in excess of 34 years. Age was significantly associated with both an FTP (Allemand et al., 2021; He et al., 2026) and a sense of purpose (Pfund et al., 2024) in a series of studies by P. L. Hill and colleagues. These considerations underscore the need for future cross-cultural and cross-occupational group research.

### Implications

The present study contributes to the literature by extending the sense of purpose theory and offering practical implications regarding the mental health of undergraduate students. With respect to the microlevel psychological processes underlying de-involution, the mechanism through which a sense of purpose negatively predicts the impact of actual-ideal self-discrepancy on individuals’ perception of involution was validated in this study. This not only expands the research on the relationship between a sense of purpose and self-management but also deepens the understanding of the link between self-discrepancy and involution perception, thereby enriching self-discrepancy theory and advancing de-involution research. Furthermore, this mediating model is moderated by the FTP, and the findings provide new evidence from an Eastern cultural context to support socioemotional selectivity theory.

The conclusions offer practical implications for the alleviation of college students’ anxiety and psychological pressure that arise from involution, thereby safeguarding healthy social mentalities and fostering a spirit of striving. According to our model decomposition results, the direct effect of sense of purpose on perceived involution accounts for 77.78% of the total effect, while the indirect mediating effect through actual-ideal self-discrepancy makes up 22.22%. The dominant share of the direct effect demonstrates that fostering adolescents’ sense of purpose is the primary intervention strategy to reduce their feelings of involution. At the same time, the relatively small mediating effect suggests that narrowing the gap between one’s ideal and real self functions as a supporting intervention path. In light of these differences in effect magnitude, we further elaborate targeted educational measures: universities can prioritize systematic courses focused on building personal purpose as the core intervention, alongside group counseling sessions that guide students to develop rational self-awareness, narrow self-discrepancy, and may collectively contribute to alleviating their perceived involution. First, guiding students to align their personal growth goals with the macro goals of the developing society may enhance their sense of purpose and engagement. Second, cultivating students’ FTPs and integrating their long-term aspirations with their short-term objectives may facilitate the development of self-management skills. Third, students should be taught to evaluate themselves accurately, expectations should be adjusted according to personal circumstances, and negative coping strategies such as “lying flat” or “goblin mode,” which might arise from excessive ideal–actual self-discrepancy or overemphasis on immediate outcomes, should be avoided.

### Limitations and future research

This study has several limitations. First, the cross-sectional design employed in this study supports an antagonistic interaction between an FTP and a sense of purpose. However, the cross-sectional design limits causal inferences about the variables’ relationships (Lewis et al., 2026). Besides, some researchers posit a causal relationship between these two variables in which an FTP may influence the sense of purpose (Pfund et al., 2022). Future research should examine the logical relationship between these factors in the de-involution process via longitudinal designs or experimental methodologies. Second, the FTP represents only one of the dimensions within a time perspective (Zimbardo and Boyd, 1999; He et al., 2024). Zimbardo and Boyd (2008) subsequently proposed the balanced time perspective theory, positing that a balanced time perspective is associated with higher levels of past positive, present hedonistic, and future-oriented tendencies in conjunction with lower levels of past negative and present fatalistic tendencies (Li X. et al., 2024). Further investigations into the ways that the other dimensions of balanced time perspective influence involution are needed. Additionally, both time perspective and a sense of purpose–along with their relationships with actual-ideal self-discrepancy–are associated with the breadth of time perception among Chinese individuals (Ji et al., 2023; Wang and Liu, 2025), and the “temporal cultural characteristics inherent in Chinese self-cognition” merit future cross-cultural comparative inquiry.

## Conclusion

Against the backdrop of widespread societal involution, this study aimed to explore the micro-level mechanism of de-involution among undergraduates. Three hypotheses were proposed accordingly: (1) sense of purpose negatively predicts perceived involution; (2) actual–ideal self-discrepancy mediates the association between sense of purpose and perceived involution; (3) future time perspective (FTP) moderates this indirect mediating pathway. The findings confirm these hypotheses. First, consistent with Hypothesis 1, individuals with a stronger sense of purpose reported lower perceived involution and less actual–ideal self-discrepancy. Second, Hypothesis 2 was supported: actual–ideal self-discrepancy played a mediating role. Effect decomposition showed that the direct effect accounted for 77.78% of the total effect, while the indirect mediating effect occupied 22.22%. Third, the data validated Hypothesis 3: the mediating effect of actual–ideal self-discrepancy was weaker for undergraduates with higher FTP compared with those with lower FTP. Overall, this study underscores the importance of cultivating a sense of purpose and FTP to facilitate de-involution and mitigate the hollowed-heart phenomenon among undergraduates.

## Data Availability

The raw data supporting the conclusions of this article will be made available by the authors, without undue reservation.
